# Comparison of Hemodynamic and Inflammatory Changes between Transoral and Transthoracic Thoracoscopic Surgery

**DOI:** 10.1371/journal.pone.0050338

**Published:** 2013-01-03

**Authors:** Yen Chu, Chien-Ying Liu, Yi-Cheng Wu, Ming-Ju Hsieh, Tzu-Ping Chen, Ying-Kai Chao, Ching-Yang Wu, Hsu-Chia Yuan, Po-Jen Ko, Yun-Hen Liu, Hui-Ping Liu

**Affiliations:** 1 Department of Thoracic Surgery, Chang Gung Memorial Hospital, Chang Gung University, Taoyuan, Taiwan; 2 Department of Thoracic Medicine, Chang Gung Memorial Hospital, Chang Gung University, Taoyuan, Taiwan; 3 Laboratory Animal Center, Chang Gung Memorial Hospital, Chang Gung University, Taoyuan, Taiwan; Roswell Park Cancer Institute, United States of America

## Abstract

**Background:**

Natural orifice transluminal endoscopy has been developed for abdominal surgical procedures. The aim of this study was to compare the surgical outcome between a novel transoral approach and a standard transthoracic approach for the thoracic cavity in a canine model.

**Methods:**

Twenty-eight dogs were assigned to transoral (n = 14) or standard thoracoscopy (n = 14). Each group underwent thoracic exploration, pre-determined surgical lung biopsy, and pericardial window creation. Blood draws were obtained before surgery and at postoperative days 1, 3, 7, and 14. Operative time, complications, laboratory parameters, hemodynamic parameters, and inflammatory parameters were compared between the two procedures. The animals were monitored for two weeks and necropsy were performed for surgical outcome evaluation.

**Results:**

The thoracic procedures were successfully performed in all of the dogs, with the exception of one animal in the transoral group. There were no serious acute or delayed complications related to surgery. There was no difference between the two surgical groups for each of the hemodynamic parameters that were evaluated. Regarding the immunological impact of the surgeries, transoral thoracoscopy was associated with significant elevations in interleukin 6 and c-reactive protein levels on postoperative days 1 and 3, respectively, when compared with the standard thoracoscopy. All dogs recovered well, without signs of mediastinitis or thoracic infection. Necropsy revealed absence of infection, no injury to vital organs, and confirmed the success of the novel procedure.

**Conclusions:**

This study suggests that both techniques were comparable with respect to procedure success rate, hemodynamic impact, and inflammatory changes. Furthermore, there was no difference in the incidence of postoperative discomfort between groups.

## Introduction

Natural orifice transluminal endoscopic surgery (NOTES) has been used as a diagnostic and therapeutic platform to perform a wide variety of abdominal surgical procedures, including peritoneoscopy, cancer staging, appendectomy, cholecystectomy, and nephrectomy [1]–[4]. By eliminating conventional abdominal incisions, several potential advantages have been proposed, including faster recovery, shorter hospital stay, less postoperative pain, better post-surgery pulmonary function, and improved cosmetic outcome. Recent reports have also confirmed the safety and benefits of NOTES in abdominal surgery in human studies [5]–[7].

The feasibility of NOTES thoracoscopy has been demonstrated in various access methods (transvesical, transesophageal, transtracheal, and transoral) in porcine and canine models [8]–[10]. However, there have been no large, randomized, controlled trials of NOTES in thoracic surgery. We previously reported the use of a transoral technique to perform thoracoscopy, pericardial window creation, and surgical lung biopsy without complications in a survival canine model [11]–[13]. To further evaluate the potential benefits of transoral thoracoscopy, we aimed to compare the surgical outcome of minor thoracic intervention using the recently developed transoral method compared with the standard transthoracic thoracoscopy in a randomized study. The experiment focused on surgical outcomes and evaluated relevant safety issues, including operative complications, cardiopulmonary impact, and inflammatory changes. Oxidative activity of neutrophils, inducible nitric oxide synthase (iNOS) expression of monocytes, and cytokine interleukin 6 (IL-6) are good guides in the assessment of the activation of the systemic inflammation to tissue injury. The expression of these mediators as well as c-reactive protein (CRP) play a major role in the acute-phase response against surgical stress [14]–[15]. They are objective and quantitative parameters in the assessment of inflammatory system activation in response to operative trauma. Therefore, these mediators were measured to clarify the surgical stress of thoracoscopy via different approaches.

## Methods

### Ethics Statement

The study was conducted in accordance with the Guide for the Care and Use of Laboratory Animals as promulgated by Institutional of Laboratory Animal Resources, National Research Council, U.S.A. The protocol of the animal experiment was approved by the Institutional Animal Care and Use Committee (IACUC) of Chang Gung Memorial Hospital in Taiwan (No. 2009121003).

### Dogs and surgical procedures

Twenty-eight dogs with a mean body weight of 8.5 kg (7.2–10.6 kg) were obtained from BioVet Beagle Farm, Pingtung, Taiwan. They were housed under standard lighting (12 hours light/dark cycle) and temperature conditions, and received humane care for the animal research at laboratory animal center of Chang Gung Memorial Hospital in Taouyan, Taiwan. All animals were fed with regular diet, and then fast overnight with unrestricted to water prior to surgery. The procedures were performed with the animals under general anesthesia using intramuscular injection of ketamine (5 mg/kg, Pfizer Inc, Taipei, Taiwan) and xylazine hydrochloric acid (HCL, 10 mg/kg). The animals were then placed in the supine position and intubated with a homemade endotracheal tube into a mainstem bronchus to provide single lung ventilation during surgery. The general anesthesia was maintained with 2% isoflurane. Before performing the thoracoscopy, an antibiotic (cefazolin, 20 mg/kg, Standard Chem & Pharm, Tainan, Taiwan) was administered for prevention of postoperative infection.

A 9 mm homemade metal tube was introduced through the vestibular incision and advanced down to the mandibular periosteum. The metal tube was advanced carefully from the premandibular space, through the pretracheal space, and into the submanubrial space. The mediastinal incision was carefully performed according to a previously reported procedure [11]–[13]. Briefly, the blunt tip of the metal tube was used to make a linear incision over the upper anterior mediastinal pleura by blunt dilation. Once the incision was complete, the metal tube was advanced into the thoracic cavity. The bronchoscope was then advanced through the metal tube for thoracic cavity exploration. For pericardial window creation, a needle knife (KD-10Q-1, Olympus Optical Co. Ltd., Tokyo, Japan) was introduced through the working channel of the flexible bronchoscope. The pericardium was incised anterior to the phrenic nerve with the tip of needle knife. The endoscope was then advanced through this window into the pericardial cavity using the needle knife as a guide, and was used to explore the pericardial cavity. For surgical lung biopsy, the predetermined lung region was resected with the electrocautery loop (Olympus Optical Co. Ltd., Tokyo, Japan) and endoscopic grasper through a 1.9 mm bronchoscopic working channel. The resected lung margin was reinforced with a homemade (Ethicon, Somerville, NJ, USA) endoloop ligature to prevent air leakage. The vestibular incision was then closed with a running 3-0 Vicryl suture (Ethicon, San Angelo, TX, USA) as previously described [11]–[13]. Soon after completion of wound closure, postoperative local anesthesia with xylocaine 20 mg was administrated over the incision. The animal was then recovered from anesthesia and returned to their cages. The arterial blood gases were analyzed immediately before and after surgery and on the 14th day.

### Care after surgery

Regular oral feeding was restarted after the animals were recovered from anesthesia. The animals were closely monitored daily for general status, eating status, social behavior, and signs of mediastinitis, sepsis, clinical signs of postoperative discomfort (decreased food intake, weight reduction, or inappropriate social behavior), and survival for 2 weeks after experiment. The animals were euthanized with xylocaine 200 mg intravenously after sedation with the ketamine and xylazine hydrochloric acid combination described above. The necropsy was performed via medial sternotomy incision and evaluated for evidence of infection, bleeding, or signs of injury to adjacent organs.

### Hemodynamic study

Immediately following intubation and then at 5 min intervals until the animal completely recovered from anesthesia, in addition to respiratory rate (breaths per minute, bpm), the values of mean arterial pressure (MAP, mmHg), heart rate (HR, beats per minute, bpm), cardiac index (CI, L/[min·m^2^]), systemic vascular resistance (SVRI, [dyn·s]/[cm^−5^·m^2^]), and global end-diastolic volume index (GEDVI, mL/m^2^) were recorded by transpulmonary thermodilution technique operated by the pulse contour cardiac output (PiCCO®) system (Pulsion Medical Systems, Munich, Germany)

### Leukocyte subset analysis and isolation of neutrophils and mononuclear cells

Total leukocyte count was measured preoperatively and postoperatively at day 1, day 3, day 7, and day 14. Specific mouse anti-canine CD3, CD4, CD8, CD14, or CD45 monoclonal antibodies (BD PharMingen™, BD Biosciences, Franklin Lakes, NJ, USA) were used to define the subpopulations of leukocytes (CD4^+^ and CD8^+^ lymphocytes, monocytes and neutrophils) by flow cytometry (Becton Dickinson, Mountain View, CA, USA).

### Intracellular oxidative activity of neutrophils

The neutrophils (2×10^5^ cells in 200 µL of supplemented RPMI medium) were supplemented with cell-permeable, fluorogenic 2′,7′-dichlorohydrofluorescein-diacetate (DCFH-DA, 5 µM; Sigma, St. Louis, MO, USA) for 30 min. Upon intracellular hydrolysis and subsequent oxidization, fluorescent DCFH was generated and analyzed quantitatively by flow cytometry.

### iNOS expression of monocytes

After cytospin preparation of peripheral blood mononuclear cells and fixation in methanol, the slides were prepared as previously described [16]. A rabbit polyclonal antibody to canine iNOS (Abcam Biochemicals, Cambridge, UK) was used for immunological staining. Antibody labeling was visualized by an avidin-biotin complex method (LSAB 2 kit, Dako, Carpenteria and Vector Laboratories, Burlingame, CA, USA). An image analyzer was used to determine the intensity of iNOS expression of monocytes.

### Enzyme-linked immunosorbent assay for IL- 6 and CRP

Serum for IL-6 and CRP analysis was stored at −80°C, and the levels of IL-6 and CRP were determined using a commercially available enzyme-linked immunosorbent assay (ELISA) kit with monoclonal antibodies specific for IL-6 (Quantikine, R & D Systems Inc., Minneapolis, Minnesota, USA) and CRP (PharMingen, BD Biosciences, San Diego, California, USA), according to the manufacturers' instructions.

### Statistical analysis

Data are presented as mean and 95% confidence interval (C.I.) except otherwise mentioned. Since the data did not approximate a Gaussian distribution (for example, the mean value did not approximate the median value), nonparametric statistical analyses were used. The Mann–Whitney *U* test was performed to assess the significance of differences between 2 groups. The frequency distributions between 2 groups were tested by using a Fisher exact probability test. GraphPad Prism (version 4.0, GraphPad Software, San Diego, CA, USA) was used for all statistical analyses. Statistical significance was defined as P<0.05.

## Results

All animals survived the follow-up period of 14 days. Thoracoscopy with surgical biopsy and pericardial window creation was successfully performed in 27 animals, with the exception of 1 animal in the transoral group. There was no different in the mean procedure time and mean weight gain between groups. None of the animals in either group had intraoperative or perioperative complications related to surgery. No postoperative complications such as infection or mediastinitis were found during the 2-week postoperative period (Table 1).

**Table 1:** Characteristics of the Experimental Animals.

Characteristics	Total	Thoracic Surgery		P value
		Transoral	Transthoracic	
Dogs (No.)	28	14	14	
Body weight (kg, mean ± SD)	8.5±1.2	8.8±1.1	8.3±1.3	0.2229
Surgical time (min, mean ± SD)	55.8±14.4	55.4±13.1	56.3±16.1	0.9632
Successful procedure	27	13	14	
Weight gain (after 14 days, kg, mean ± SD)	0.33±0.53	0.20±0.59	0.46±0.13	0.1956
Postoperative complications (No.)				
Fever	6	2	4	0.6483
Infection or mediastinitis	0	0	0	
Pain	4	2 (1 mild, 1 severe)	2 (severe)	1.0
Necropsy after 14 days (No.)				
Infection	0	0	0	
Organ injury	0	0	0	
Correct lung biopsy	27	13	14	1.0
Good healing of pericardial window	17	8	9	1.0
Local adhesion	25	12	13	1.0

### Temperature

Six animals showed rectal temperature elevation after surgery. In 2 dogs from the transoral group, the temperature rose above normal on day 1–3 and returned to normal 7 days after surgery. In the transthoracic group, 4 animals had elevated temperatures at 3–7 days after surgery, which returned to baseline at 2 weeks in 3 of the animals (Table 1).

### Hemodynamic study

**Figure 1:**
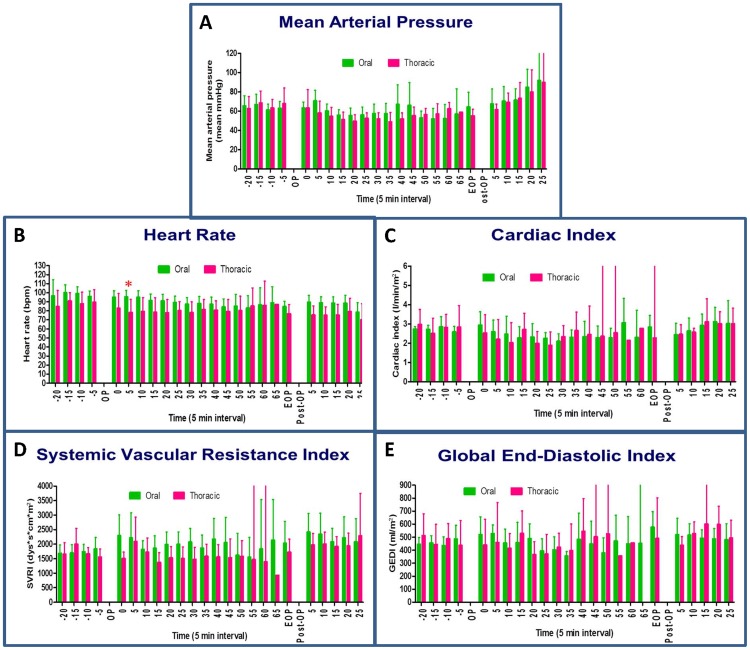
Hemodynamic analyses, including mean arterial pressure (A), heart rate (B), cardiac index (C), systemic vascular resistance index (D), and global end-diastolic index (E). N = 12 for the transoral approach and n = 12 for the transthoracic approach. *P<0.05, transoral vs. transthoracic approach for the same time interval, by Mann–Whitney *U* test.

Comprehensive hemodynamic changes were recorded in 24 animals. We observed a significant decline in HR and a moderate variation in CI during surgery. We also observed a minor decrease in GEDI and MAP compared to baseline. However, the change in the cardiopulmonary parameters between the groups was not statistically significant, and no clinically relevant immediate or late complications were identified after surgery (Fig. 1).

### White blood cells

**Figure 2:**
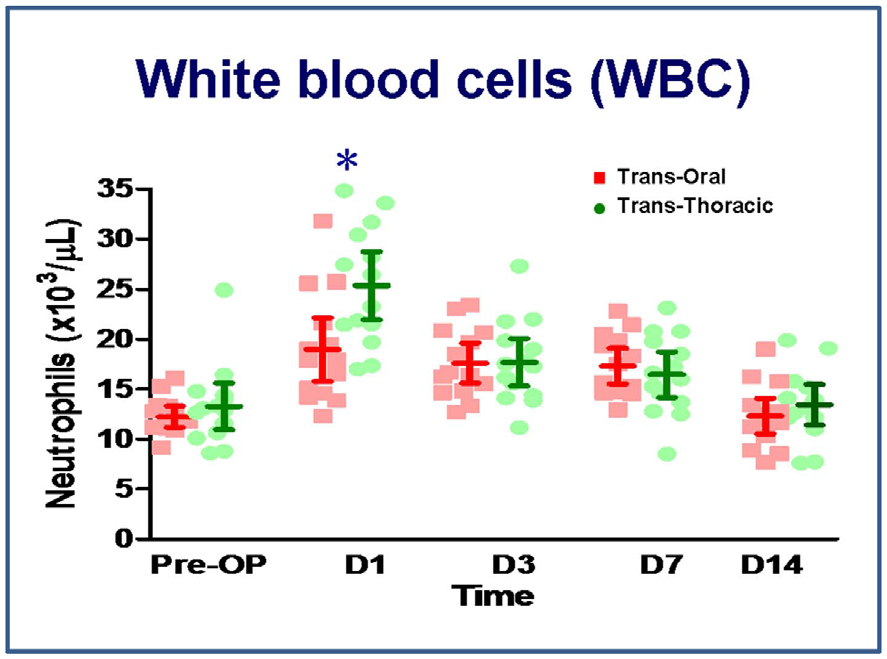
White blood cell counts. N = 14 for the transoral approach and n = 14 for the transthoracic approach. *P<0.05, transoral vs. transthoracic approach for the same time point, by Mann–Whitney *U* test.

Among the 28 animals, 25 animals showed an increased white blood cell count above baseline 1 day after surgery, which included 15 animals from the transthoracic group and 10 from the transoral group. The white blood cell (WBC) count returned to baseline in 24 of the 28 animals at 2 weeks. The WBC count of transthoracic group was significantly higher on day 1 after surgery relative to the transoral group, but there was no significant difference in WBC count between the groups thereafter (Fig. 2).

### Neutrophils and oxidative activity

**Figure 3:**
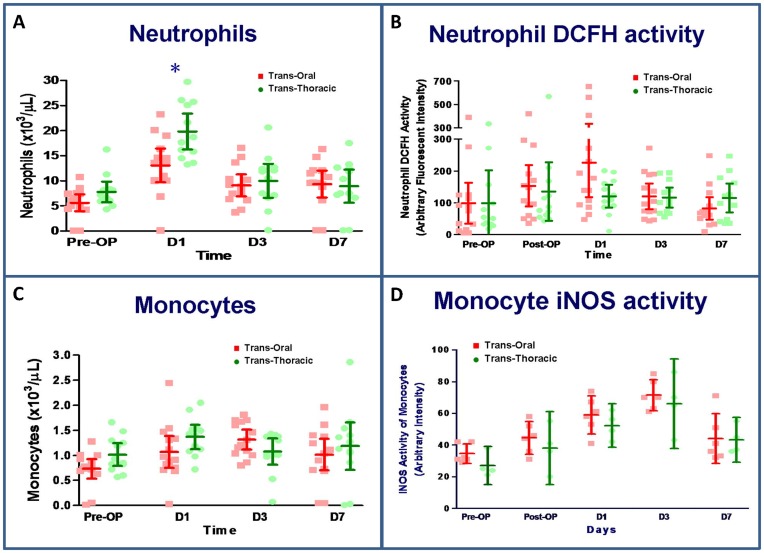
Neutrophil cell counts (A) and dichlorodihydrofluorescein (DCFH) activity (B) as well as monocyte cell counts (C) and iNOS activity (D). N = 14 for the transoral approach and n = 14 for the transthoracic approach. *P<0.05, transoral vs. transthoracic approach for the same time point, by Mann–Whitney *U* test.

The number of neutrophils was elevated on postoperative day 1, 3, and 7 in both groups. This variation was significantly increased in the transthoracic group relative to the transoral group on the first postoperative day. Neutrophil activation was revealed by expression of DCFH, which increased significantly in both groups on postoperative day 1 and returned to preoperative levels at 1 week. However, DCFH expression was not significantly different between the groups (Fig. 3A and 3B).

### Monocytes and the intensity of iNOS expression

During the postoperative period, the number of monocytes increased significantly in both groups following surgery, and remained elevated above the baseline until 7 days post-surgery. Significantly increased iNOS levels were observed in both groups during the postoperative period; however, there were no statistically significant differences between the transoral and transthoracic groups with regard to monocyte cell counts and iNOS expression (Fig. 3C and 3D).

### Lymphocytes and CD4/CD8 T lymphocyte subset analysis

**Figure 4:**
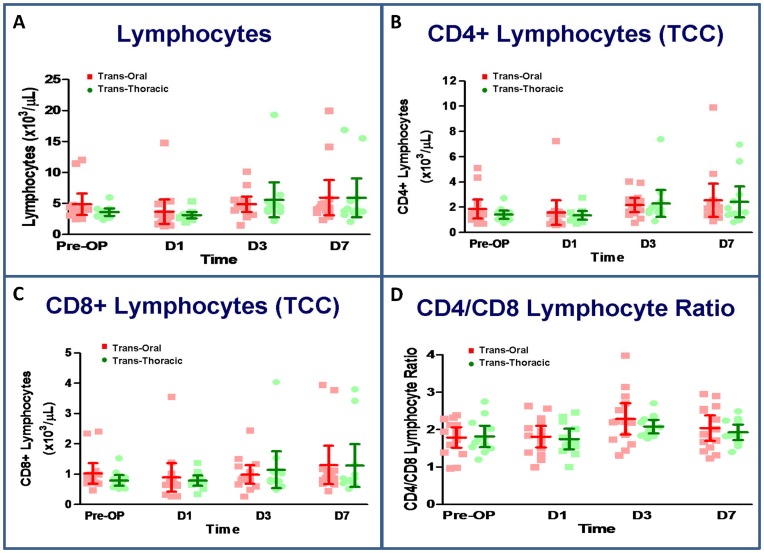
Lymphocyte cell counts (A), the CD4 (B) and CD8 (B) subsets, and CD4/CD8 lymphocyte ratio (D). N = 14 for the transoral approach and n = 12 for the transthoracic approach. *P<0.05, transoral vs. transthoracic approach for the same time point, by Mann–Whitney *U* test.

There was a significant decrease in the total lymphocyte count on postoperative day 1 in both groups. Thereafter, the total lymphocyte count increased significantly above the baseline on postoperative day 3. With regard to T lymphocyte subsets, the number of CD4^+^ T cells increased on postoperative days 3 and 7, the number of CD8^+^ T cells did not differ significantly from preoperative levels at any of the time points throughout the study, and a significant elevation in the CD4∶CD8 ratio was also noted on postoperative day 3 in both groups. However, there were no significant differences in total lymphocyte count, T lymphocyte subsets, or CD4∶CD8 ratio between the groups at any of the study time points (Fig. 4).

### Interleukin 6 and C-reactive protein levels

**Figure 5:**
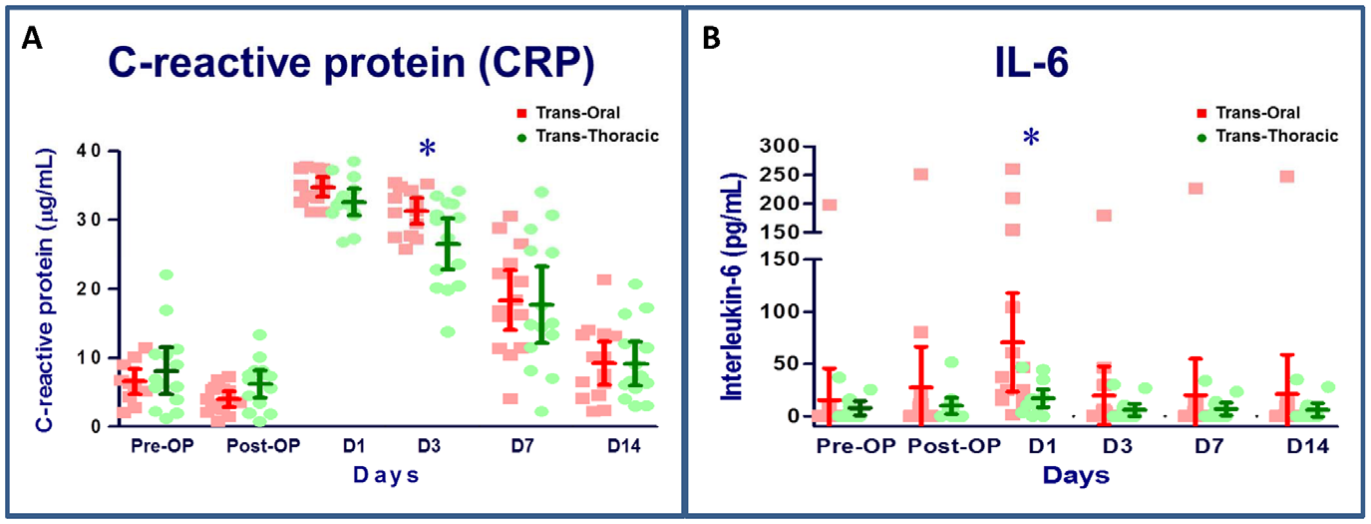
Serum levels of c-reactive protein (A) and interleukin 6 (B). N = 14 for the transoral approach and n = 14 for the transthoracic approach. *P<0.05, transoral vs. transthoracic approach for the same time point, by Mann–Whitney *U* test.

Plasma levels of CRP increased significantly 1 day after surgery in both groups. The CRP levels were significantly higher in the transoral group compared with the transthoracic group at day 3. However, there was no group difference in the plasma levels of CRP on day 7 and day 14. The serum concentration of IL-6 was increased over preoperative levels in early postoperative period in both groups. This elevation in IL-6 levels was greater in the transoral group than in the transthoracic group at day 1 after surgery. However, no differences were observed between the groups 3 days after surgery (Fig. 5).

### Arterial blood gas

**Figure 6:**
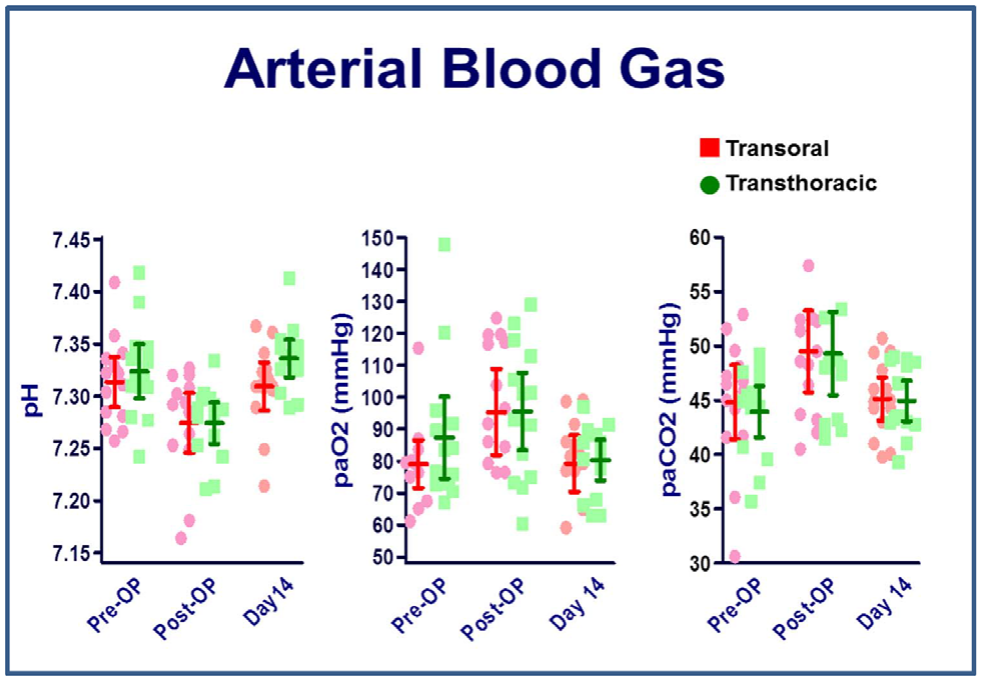
Arterial blood gas analysis. N = 14 for the transoral approach and n = 14 for the transthoracic approach. *P<0.05, transoral vs. transthoracic approach for the same time point, by Mann–Whitney *U* test.

There was no significant change in PaO2 values before and after the procedure. The pH value was decreased and PaCO2 level was elevated immediately after the procedure. Both values returned to the pre-operative levels before day 14 post-operatively. There was no difference in the values of pH, PaO2, and PaCO2 between the two approaches at the same time point (Fig. 6).

### Postoperative pain

In the study animals, postoperative discomfort manifested as decreased food intake, weight reduction, or inappropriate social behavior after surgery; 4 of 28 dogs presented with moderate to severe postoperative wound pain. There was no significant difference in the severity of pain between the groups (1 animal with mild pain and 1 animal with severe pain in the transoral group vs. 2 animals with severe pain in the transthoracic group; Table 1).

### Necropsy

At the time of necropsy, there were no signs of infection or evidence of organ injury in either the thoracic cavity or the mediastinum. The lung biopsy was correctly performed in 27 out of the 28 animals (n = 13 in the transoral group, n = 14 in the transthoracic group, P = 1.0). The pericardial window had obliterated and the surgical wounds were difficult to identify in 17 out of the 28 animals (n = 8 in the transoral group, n = 9 in the transthoracic group, P = 1.0). Twenty-five animals revealed adhesions either on the pleural or mediastinum at the surgical lung biopsy region (n = 12 in the transoral group, n = 13 in the transthoracic group, P = 1.0; Table 1).

## Discussion

We recently published a study demonstrating the successful performance of a minor thoracic procedure via a transoral approach in a canine model [11]–[13]. In line with these findings, our present study further demonstrated the safety and efficacy of the transoral approach in hemodynamic, inflammatory conditions. These findings are clinically relevant, as our study included thoracic exploration, surgical lung biopsy, and pericardial window creation in a survival canine model, and each of these procedures is commonly performed in daily practice.

Since Kallo et al. reported the first transgastric peritoneoscopy in 2004, NOTES has been increasingly used in the diagnosis and therapy of both benign and malignant disease of the abdomen [17] Natural orifice surgery (NOS) has potential advantages over conventional laparoscopic surgery; however, the development of NOTES in thoracic surgery was delayed. The critical problem that hindered the implementation of NOTES in thoracic disease was the lack of a reliable platform for approaching the thoracic cavity via a natural orifice. Furthermore, potential complications during the procedure, including incision leakage, mediastinal organ injury, and infection were important concerns.

Several studies have investigated the feasibility of using the natural orifice as a novel platform for thoracic intervention and emphasized that an optimal approach was key to successful natural orifice thoracic surgery. Lima et al. reported their experience with 6 pigs that underwent transvesical lung biopsy. Their overall success rate was 100%. The authors found that a major disadvantage of this intervention is difficulty in performing the procedure over the upper mediastinum and pleural region [8]. Palma et al. reported another study of 4 pigs that underwent transgastric lung biopsy. Their success rate was 100%; however, results from a few of the experimental animals failed to effectively assure the safety of a transgastric approach [18]. Sumiyama and colleagues performed transesophageal thoracoscopy in 9 pigs and demonstrated an increased risk of tension pneumothorax and massive bleeding [19]. Our group previously described the utilization of transtracheal thoracoscopy in 12 dogs; we encountered 3 cases of tension pneumothorax due to inadvertent injury to the lung during creation of a tracheal access site [20]. Recently, we have made significant progress in using a transoral route for surgical lung biopsy and pericardial window creation, without major intraoperative or postoperative complications [10]–[13]. Our findings suggest that a transoral approach can be used as a novel platform for studies of NOTES for intrathoracic surgery. However, its clinical application has yet to be implemented, as there is uncertainty regarding the impact of hemodynamic and inflammatory status during the procedure.

In 2011, our research group showed that identification of the anterior medial aspect of the thoracic cavity is possible with the transoral approach, and creation of a pericardial window and surgical lung biopsy is technically feasible in a canine model [11]–[13]. In this comparative study, 93% of the target lung regions were successfully accessed by the present approach. We expect that the present transoral method will play a central role in the development of NOTES in intrathoracic surgery. The safety of novel surgical procedures is one of the most critical barriers to gaining acceptance in clinical practice. Our previous study demonstrated that 90% to 95% of surgical lung biopsies could be completed by using the transoral route without major complications. The present study also demonstrates that using the transoral route for thoracic intervention is safe and practical in a canine model. This method did not cause infection or injury to the vital organs in all 14 animals, suggesting that the use of transoral thoracoscopy is a potential alternative to the current transthoracic endoscopic method, especially in young adults that are concerned about postoperative cosmesis.

Many recent reports in the literature regarding the hemodynamic impact of NOTES during surgery have been studied in a porcine model. In 2011, Suzuki et al. evaluated the cardiopulmonary effect of transvaginal NOTES cholecystectomy in 10 pigs [21]. This study demonstrated that there was similar cardiopulmonary stability in both the transvaginal NOTES and the laparoscopic cholecystectomy groups. With regard to cardiopulmonary safety of NOTES in intrathoracic surgery, Von Delius et al. studied hemodynamic impact in 8 pigs that underwent transesophageal mediastinostomy [22]. They observed hemodynamic instability in 3 animals, due to an injury in the parietal pleura, which resulted in tension pneumothorax and mortality in 1 pig. These results highlight the importance of using a safe and effective surgical platform and monitoring the hemodynamic status during NOTES. Our current study revealed a similar impact of hemodynamic status in the transoral and standard transthoracic approaches in a comparative animal study, which suggests that transoral access to the thoracic cavity is a safe platform compared with the standard transthoracic approach.

When the clinical application of the novel platform is considered, the invasiveness of the procedure is another major concern that must be addressed. Several researchers have evaluated inflammatory and immunologic responses in both animal and human studies following endoscopic and open surgery, and suggest that endoscopic surgery is associated with less surgical stress than the open surgical approach [14]–[15]. In the current study, the transthoracic group showed higher IL-6 and CRP levels than the transoral group. However, only 1 of 6 IL-6 and CRP measurements was found to be significantly different. Furthermore, there were no clinically apparent complications in both groups. Regarding the cellular responses to the surgical procedure, we found a similar change in iNOS, DCFH, and CD4/CD8 indicators in the transoral group compared with those that underwent conventional thoracoscopy. Based on these results, our study suggests that transoral thoracoscopy results in similar surgical stress as the current transthoracic approach. Additionally, the arterial blood gas analysis revealed no difference between groups, suggesting that transoral thoracoscopy did not increase pulmonary dysfunction, compared to the transthoracic approach.

The present transoral method offers several potential advantages over the transthoracic technique that is currently in use. First, the technique demonstrated good cosmetic outcomes, as the surgery was completed without any external thoracic incision. Second, the transoral group experienced similar acute postoperative pain compared with the animals undergoing transthoracic surgery. However, many studies reported that thoracic incisions may result in post-thoracotomy discomfort and intercostal neuralgia, which can be avoided by using the transoral approach.

The present study has some limitations. First, our transoral approach for thoracic cavity techniques in a canine model may face difficulty in clinical practice, particularly in obese patients with a thick or short neck. Second, a relatively small sample size was obtained for the cellular immunity study, which may contribute to the non-significant difference observed in the cellular immune reactions between the groups. Third, we were unable to measure the complete cardiopulmonary data that were influenced by arterial line dysfunction. Fourth, since the IL-6 and CRP assays were performed 1 day after surgery in our study, we could not explore the change in inflammatory cytokine secretion immediately after surgery. Finally, we only performed the surgical lung biopsy over the anterior aspect of thorax. Thus, we cannot determine whether approaching the posterior thorax under the decubital position would be successful. Nevertheless, we designed the present experiment to be as relevant to surgical practice as possible, and in clinical daily practice surgical lung biopsy is one of the most commonly performed endoscopic procedures in thoracic surgery.

In summary, this study revealed that utilizing a transoral approach for thoracic cavity procedures was feasible, efficacious, and safe in a canine survival study. We also found that the immunologic, inflammatory, and hemodynamic responses to the transoral approach were similar to those of the conventional thoracoscopic approach, with the exception of minimal differences in inflammatory reactions. We conclude that clinical trials are needed to further clarify the clinical role of NOTES in thoracic disease.
